# Separability of motor imagery of the self from interpretation of motor intentions of others at the single trial level: an EEG study

**DOI:** 10.1186/s12984-017-0276-4

**Published:** 2017-06-26

**Authors:** João Andrade, José Cecílio, Marco Simões, Francisco Sales, Miguel Castelo-Branco

**Affiliations:** 1 IBILI – Institute for Biomedical Imaging in Life Sciences, Faculty of Medicine, University of Coimbra, Coimbra, Portugal; 2 CIBIT, ICNAS, Brain Imaging Network of Portugal, Coimbra, Portugal; 3 CISUC, Faculty of Sciences and Technology, University of Coimbra, Coimbra, Portugal; 4 Coimbra University Hospital, Coimbra, Portugal

**Keywords:** EEG, Motor imagery, Classification, Single trial

## Abstract

**Background:**

We aimed to investigate the separability of the neural correlates of 2 types of motor imagery, self and third person (actions owned by the participant himself vs. another individual). If possible this would allow for the development of BCI interfaces to train disorders of action and intention understanding beyond simple imitation, such as autism.

**Methods:**

We used EEG recordings from 20 healthy participants, as well as electrocorticography (ECoG) in one, based on a virtual reality setup. To test feasibility of discrimination between each type of imagery at the single trial level, time-frequency and source analysis were performed and further assessed by data-driven statistical classification using Support Vector Machines.

**Results:**

The main observed differences between self-other imagery conditions in topographic maps were found in Frontal and Parieto-Occipital regions, in agreement with the presence of 2 independent non μ related contributions in the low alpha frequency range. ECOG corroborated such separability. Source analysis also showed differences near the temporo-parietal junction and single-trial average classification accuracy between both types of motor imagery was 67 ± 1%, and raised above 70% when 3 trials were used. The single-trial classification accuracy was significantly above chance level for all the participants of this study (*p* < 0.02).

**Conclusions:**

The observed pattern of results show that Self and Third Person MI use distinct electrophysiological mechanisms detectable at the scalp (and ECOG) at the single trial level, with separable levels of involvement of the mirror neuron system in different regions.

These observations provide a promising step to develop new BCI training/rehabilitation paradigms for patients with neurodevelopmental disorders of action understanding beyond simple imitation, such as autism, who would benefit from training and anticipation of the perceived intention of others as opposed to own intentions in social contexts.

## Background

Motor imagery (MI) is defined as a dynamic state during which a subject simulates an action mentally irrespective of body movement [1]. This mental simulation can be subdivided into third person and self imagery [2]. Third person imagery corresponds to the imagery of the movement performed by another person, while self imagery concerns the person imaging himself/herself performing a movement. MI of the self or others involves constructing a mental internal representation of an action. The observation of a motor act (e.g. grasping an apple), done by another individual, allows one to extract information about the goal (grasping) and its underlying intention (e.g. grasping for eating).

Imagery may involve multiple types of strategy, and probably implies different ability levels to elaborate vivid constructs. Poor imagers may need additional recruitment of particular brain systems such as the cortico-striatal and cortico-cerebellar networks, which does not occur when skilled imagers perform this type of task [3]. There is substantial evidence [4] showing that performance in imagery tasks improves with practice.

According to previous reports, MI and motor performance (MP) share part of the same neural networks [5]. The networks involved in both MI and MP include the contralateral motor, premotor cortex and homotopic ipsilateral regions [6–8]. Olsson, CJ et al. (2008) [9] suggest, in line with the above mentioned ideas, that the knowledge of the networks involved in MI may be used for skill enhancement in sports and in the design of brain-computer interface (BCI) paradigms for gaming purposes [10]. In fact, the strong overlap between MI and MP brain processes may be used to help in rehabilitation therapies for movement recovery in stroke patients using imagery targeted at movements that need to be rehabilitated [11, 12].

Several studies performed in macaques and in humans [13–15] showed the existence of so-called mirror neurons that fire not only when a given agent performs an action of his/her own but also when others are observed performing the same action. The studies performed in macaques identified mirror neurons in two anatomically connected cortical areas: the posterior part of the inferior frontal cortex and the anterior part of the inferior parietal lobule. These parts constitute the fronto-parietal mirror-neuron system (MNS) [13].

Concerning studies performed in humans, a seminal functional Magnetic Resonance Imaging (fMRI) study on the imitation of finger movements showed two cortical areas, the pars opercularis of the inferior frontal gyrus and the rostral part of posterior parietal cortex, related to MNS that match the anatomical location of monkey mirror neuron areas [16].

The MNS is important because it is also relevant to studies of social cognition processes such as action and intention understanding in the context of the interpretation of social interactions, and in particular empathy and other cognitive processes beyond imitation. Accordingly, several reports [14, 17] suggested that dysfunction of the MNS in humans might represent a core deficit in neurodevelopmental disorders. Autistic subjects have difficulties in switching between self and other intentional representations [18]. This is for example instantiated in the clinical setting by the early developmental difficulty in this condition in stating the first person and using instead the third person. Having a BCI training this discrimination might help improve this neurobiological and clinical feature. Joint attention deficits are precisely related with the difficulty in encoding the intentions of others [19–21]. In other words, given that autism is a disorder of action and intention understanding proof of concept designs in this area are much needed.

Social perception and imagery related to movement performed by a third person in relation to the MNS are intensively being studied also in the context of normal cognition. Filimon et al. (2007) [22] suggest that the pattern of MNS activation is specific for the type of hand action performed (grasping vs reaching, for instance) and Taube et al. (2015) [23] studied the association between action observation and motor imagery in brain regions relevant for the control of balance. However, few studies [24] focused on the differences related to Self Motor Imagery (including Kinaesthetic components) and Third Person Motor Imagery of Other Agents (SMI and TPMI). Such a distinction goes clearly beyond simple imitation. Neuper et al. (2005) [24] found significant differences in the classification between the two imagery tasks vs baseline (but direct task comparisons were not attempted). They claim that the classification accuracy of recognition of SMI is higher than TPMI (although rates were relatively low even when classifying against baseline: 67% vs 56%). If this is true first person paradigms may be more powerful for certain motor-imagery-based BCI applications but not necessarily so in applications where third person social representations are more relevant.

Although SMI and TPMI networks share similar neural correlates, some relevant differences have been identified [5, 25]. SMI overlaps substantially with MP, except in the functional connectivity between association motor areas and superior parietal lobule with M1. However, connectivity patterns in TPMI tasks seem to be biased towards visual areas. As evidence in line with this notion, when a visual imagery condition is contrasted to a (self) Kinaesthetic condition, Guillot et al. (2009) [25] found selective action in the superior parietal lobule and occipital cortex.

In this work we aimed at investigating the neural correlates of the differences between third person and self imagery at the scalp level and the potential involvement of MNS in those tasks. In order to understand the neural networks involved, we performed an EEG study where third person and self imagery tasks were undertaken by a set of healthy participants. Time-frequency analyses and statistical classification of single or few trials, which is critical for BCI applications, were done in order to discriminate which task (centered on self or third person movement) was performed by each participant.

To get additional insight into these differences, we also had the opportunity to acquire and analyse data using the same task during electrocorticography (ECoG) in one participant.

Our results suggest that networks subserving each type of movement representation are separable at the single trial and individual scalp level, involving distinct parts of the action and intention understanding circuitry related to the MNS and beyond this system. We believe that this work provides also a potential step to develop new training/rehabilitation paradigms for people with neurodevelopmental disorders, in particular in autism, where training and anticipation of the intention of others beyond simple imitation is very relevant.

## Methods

### Study procedures

**Fig. 1:**
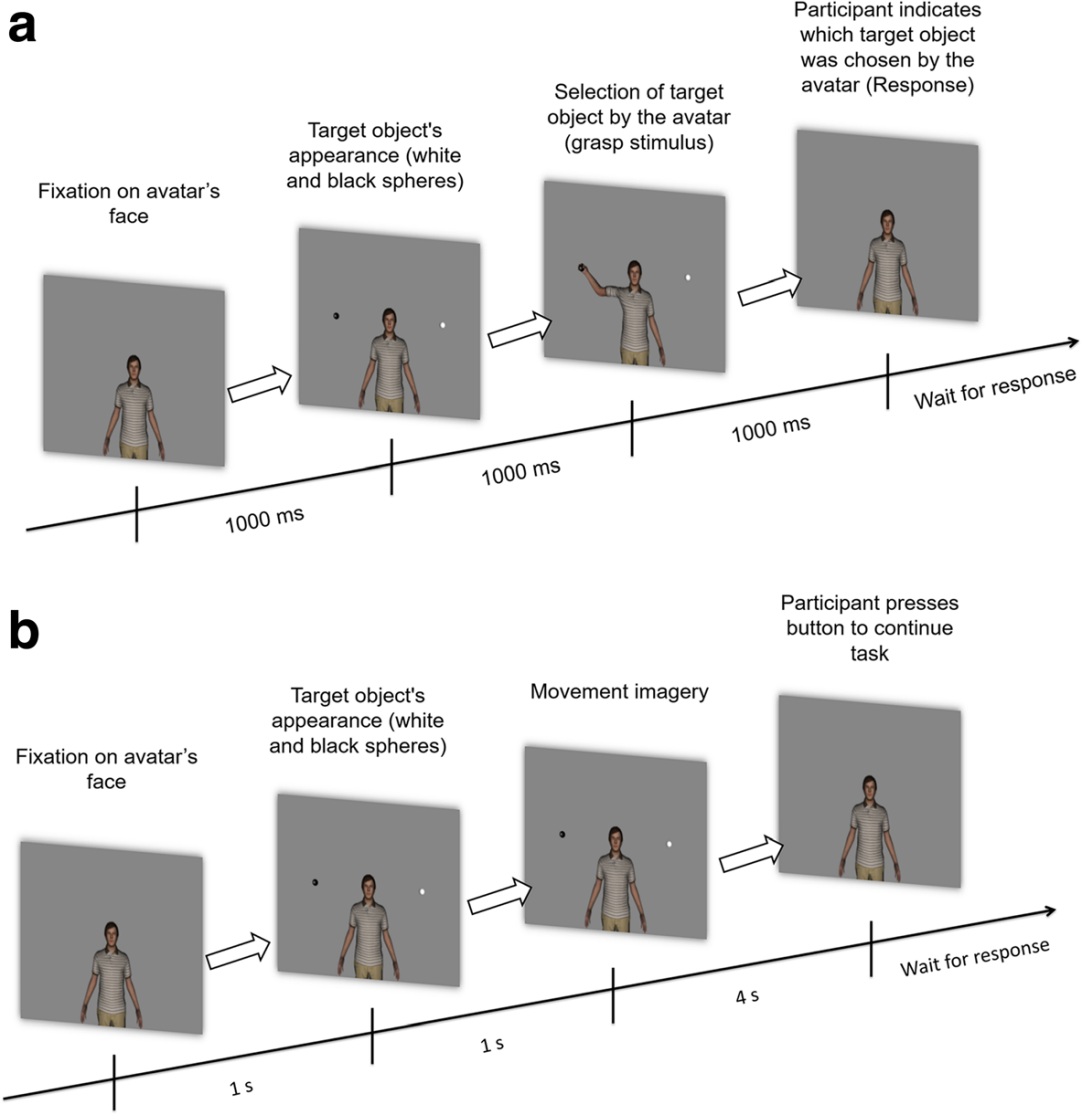
**a** Visual stimulation paradigm; **b** Imagery paradigm . Grasp experiment sequence: the imagery sequence is composed by either two different upper-limb movements (right or left arm) in a pseudo-random balanced order. The same setup is used for both SMI (self imagery and TPMI (third person imagery)

We designed a visual experimental paradigm based on an avatar and two 3D virtual “balls” placed side-by-side to the head of the avatar at arm distance, using the Vizard Virtual Reality Toolkit from WorldViz (Fig. 1). The avatar reaches for the ball positioned either on his left or right side, with his left or right hand, respectively.

Participants were first asked to observe the visual stimulus of Fig. 1, for 5 min (familiarization period) that preceded the beginning of the recordings. In this familiarization period there is a baseline of 1 s where the avatar is looking directly at the subject. The spheres (one black and one white) appear randomly on both sides of the avatar, remaining there during 1 s. The avatar reaches for one sphere randomly, grabs it, and returns to the initial position. The movement to grab the object is also performed in 1 s. Both spheres disappear after the avatar reaches its initial position. This visual stimulus was used to instruct the participants how the imagery task should be performed.

Afterwards, following a resting period, the imagery task was performed. This task was divided between two types of motor imagery (grouped by blocks of 30 trials): imagery of the avatar grasping the black sphere (third person) and imagery of the participant himself doing it (self). Each trial is composed by the appearance of the two spheres (1 s), followed by a 100 ms beep and an imagery period of 4 s. Once the 4 s had elapsed, the spheres disappeared (while maintaining the avatar on screen) and the participant had to push a button to proceed to the next trial. The button allowed the participant to control the level of comfort with the experiment. Participants were told to avoid moving and swallowing during the imagery period but they could relax from this rule before pressing the button to start a new trial. Participants were also instructed to keep their gaze at the centre of the face of the avatar.

In the EEG acquisitions, this sequence is done at least 90 times per imagery condition (SMI and TPMI), half for left and half for right side movement to ensure balancing and avoid lateralization issues. Small breaks were taken, allowing the participant to relax during the experiment. Due to the longer preparation needed in the ECoG acquisition, only about 30 trials per imagery condition were acquired.

### Participants

For the EEG study, twenty participants (19 right-handed and one left-handed healthy male adults) aged 22–34 (average 26) years old with no history of neurological or motor disorders and free of any type of injury participated in the study. One participant (female, 18 years old), undergoing ECoG monitoring due to epilepsy, able to execute simple behavioural tasks and to follow instructions performed the task and ECoG was acquired after the training trials.

An informed consent was read and signed by each participant before the start of any experiment. The study was conducted according to the approval by the Ethics Commission of the Faculty of Medicine of the University of Coimbra and the FP7 BRAINTRAIN project ethics rules.

All participants had normal or corrected-to normal vision and they were naive regarding the purpose of the study.

### Data acquisition

Participants were sit at about 70 cm from the screen (22-in. LCD Monitor; frame rate of 60 Hz, 1680 × 1050 resolution), and the EEG and EOG signals were recorded using a 64 set of electrodes from Brain Products.

Regarding the EEG acquisitions, the participant‘s scalp was first cleaned using Nuprep gel and alcohol, and then the actiCAP cap was placed on their heads. The data were acquired using BrainVision Recorder software from all 64 Ag/AgCl active electrodes (Brain Products, Munich, Germany), placed over the scalp according to the locations of international 10–10 standard system, where the position of electrodes Fp1, Fp2, FT9 and FT10 was adjusted to measure EOG signals. Those electrodes were placed around the eyes. Two electrodes were placed above and under the left eye (vertical movements and blink control) and the other two electrodes were placed on the outer side of each eye (horizontal movements control).

The ground electrode was placed at the Fpz position and two reference electrodes (originally on AF7 and AF8 positions) were used, placed at both ear lobes. Their impedance was kept lower than 15 KΩ. The electrodes were connected directly to the Brain Products’ actiCHAMP Amplifier and sampled at 1000 Hz. EEG and EOG data were recorded using the Recorder software with a notch filter at 50 Hz.

**Fig. 2:**
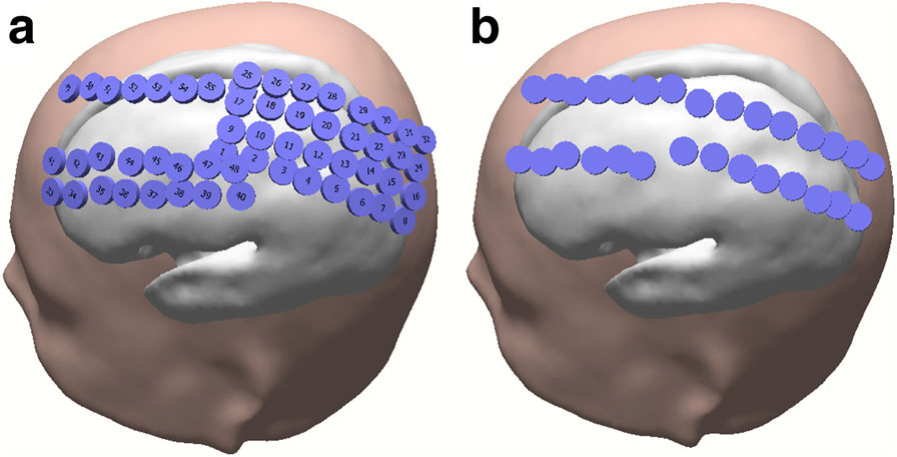
**a** ECoG with 56 electrodes grid; **b** 29 electrodes used for signal processing

The ECoG signal was acquired on 56 locations with a sampling frequency of 5000 Hz, using Neuroscan SynAmps^2^. The electrode grid was located on the left hemisphere on the locations shown on Fig. 2.

### Signal pre-processing

All the data analysis, filtering, segmentation, data cleaning (discarding of bad data segments), classification and cross-validation procedures were performed offline using custom software written in MATLAB R2013a (Mathworks Inc., Natick, MA). Filtering, segmentation and data cleaning inspection were performed using the EEGLab Toolbox v13.4.4b (Swartz Center for Computational Neuroscience, University of California, San Diego, https://sccn.ucsd.edu/eeglab/) [26] for MATLAB.

EEG data were filtered with a band-pass filter between 1 Hz and 100 Hz. Data segmentation was performed in epochs of 5 s, with 1 s before the imagery period (before the GO signal beep) and 4 s for the imagery period (after the GO signal beep). A baseline correction procedure was done using the first pre-stimulus 500 ms, as applied in [27]. Segments contaminated with eye blinks or muscular artifacts with considerable amplitude were excluded from the analysis (a threshold of 50 μV was used), resulting in, at least, 75 valid segments for each third person and self motor imagery conditions.

In order to remove noisy components from the signal, an ICA algorithm was run and a visual inspection of all components was done to remove the ones related to localised noise (electrode related, for instance) as well as ocular movement related artifacts [28].

Upon conclusion of data pre-processing, a time-frequency group analysis using paired t-tests (available in the *study* section of EEGLab Toolbox, with correction for multiple comparisons at the cluster level) was performed [29], comparing the third person and self motor imagery conditions.

In order to identify the imagery condition of the participant in a data driven manner, a classification approach based on Support Vector Machines (SVM) was performed in MATLAB using the standard algorithm from the Statistics Toolbox, configured with the linear kernel. The classification was performed using a five-fold cross-validation approach. Both training and test data were subjected to a band-pass filter between 8 and 13 Hz, because this band encompasses both α (of occipital and/or frontal origin) and μ-rhythm frequencies. The latter are well known to undergo an Event Related Desynchronisation (ERD) when a person imagines or performs a movement [30] (for analysis of other bands, see below).

After band-pass filtering, training data were subjected to a Common Spatial Pattern (CSP) algorithm and the five patterns with higher differences in variance (more significant channels) were kept. The information taken from the CSP algorithm on the training data was then applied on the test data [31].

The feature extracted from the dataset was the mean power of the signal, in the frequency domain, in the low α to μ-rhythm frequency bands, calculated according to eq. 1, where “signal” refers to task period between 500 ms after the trigger beep and 3.5 s after that beep. “Baseline” represents the 500 ms immediately before the imagery trigger beep.

1 $$ \overline{P}=\frac{\sum_{f=8}^{13} fft{\left( signal(t)\right)}^2}{13-8}-\frac{\sum_{f=8}^{13} fft{\left( baseline(t)\right)}^2}{13-8} $$

We also applied the steps described by [32] with similar results. Besides different algorithms, we also applied the classification methods in different frequency bands. For instance, some electrodes showed interesting differences in 15–17 Hz. However, this lower frequency band we present in this paper (8–13 Hz) was the one that showed higher and more significant differences, suggesting that imagery aspects not directly related to motor function (low α) are also relevant and together with μ-rhythm frequencies represent the main focus of this paper.

These features were subjected to a Principal Component Analysis (PCA) algorithm and the principal components with explained variance larger than 5% were used as features for the classifier.

**Fig. 3:**
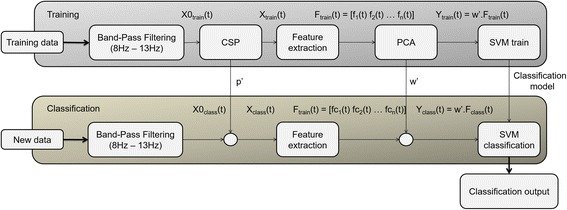
Classification algorithm diagram

The information extracted from PCA and SVM was used on the treated test dataset and the classification output was stored and analysed. Figure 3 represents a diagram flow of the processing approach.

**Fig. 4:**
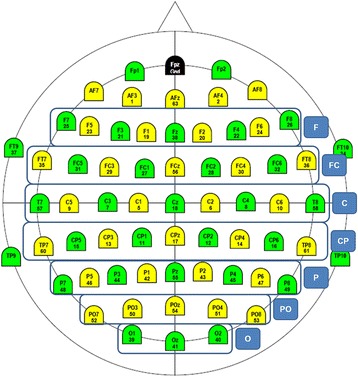
Lines of electrodes for best cluster survey

In order to understand which electrodes or clusters of electrodes would give the best results, and in order to plan further data reduction, we first conducted an exploratory survey over all channels. Taking into account the data collected from each participant, the classification results were acquired using the following 8 cluster types:

- All electrodes in the scalp;
- Seven separated lines of electrodes as represented in Fig. 4.

The cluster used to determine the participant’s discrimination accuracy was the one that presented the highest accuracy value. This survey is important because for a clinical application, the lower the number of electrodes, the better.

Finally, the statistical significance of the classification was assessed by permutation tests [33–35], using 2000 permutations.

Differences between both conditions at the group level were also assessed using the Matlab function for the Wilcoxon signed-rank test [36, 37], for eight clusters of electrodes (AF, F, FC, C, CP, P, PO and O). The same differences were also assessed subject wise using Matlab’s function for the Wilcoxon rank sum test.

The ECoG signal was resampled to 512 Hz and the pre-processing was done with a pipeline similar to EEG analysis, except for ICA because data from ECoG is much cleaner, not presenting as much noise as raw EEG data.

**Fig. 5:**
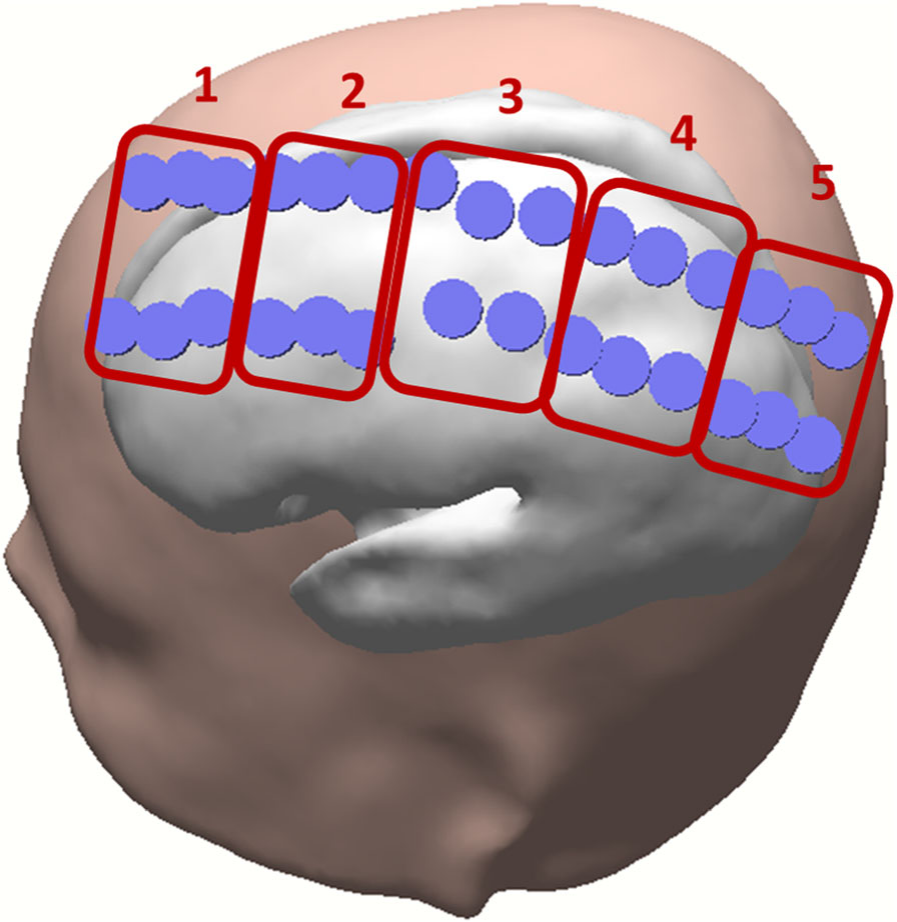
Lines of clusters (identified by number) used in the ECoG analysis

Contrarily to EEG data analyses, ECoG was divided into SMI and TPMI left and right imagery, because the grid was located in only one hemisphere and we intended to understand if laterality was implicated in this case. The minimum number of valid trials per condition achieved was 5 and maximum was 9. We performed statistical analysis using bootstrap technique [38] (2000 resamples) and Wilcoxon rank sum test on the 5 clusters presented in Fig. 5.

### Source analysis

Source analysis was applied to the EEG data, using sLoreta software package in order to assess the differences between SMI and TPMI (University Hospital of Psychiatry, Zürich, Switzerland; http://www.uzh.ch/keyinst/loretaOldy.htm). This software provides source localization of neural generators of brain electrical activity and tests for multiple comparisons [39]. Since both tasks present significant brain activity in both hemispheres, it would be important to take into account the laterality of motor imagery. In order to do that, our null hypothesis for the statistical test for source analysis is shown on eq. 2.

2 $$ {TPMI}_L-{TPMI}_R={SMI}_L-{SMI}_R $$

TPMI_L_ and TPMI_R_ represent the imagery of the left and right (respectively) upper limb of the avatar; SMI_L_ and SMI_R_ means the imagery of the participants own left and right (respectively) upper limb. Results were acquired using subject-wise data normalization and paired statistics for the imagery period in the same frequency interval used for classification and other analyses (8–13 Hz).

## Results

We first describe the neural representation of third person and self motor imagery (TPMI and SMI), as well as their their respective differences. We then investigate the neural underpinnings of such differences by means of source analysis. Finally we show that automatic classification significantly differentiates between these tasks, and further highlight their neural underpinnings using ECOG.

### The neural representation of third person motor imagery (avatar grasping a sphere)

**Fig. 6:**
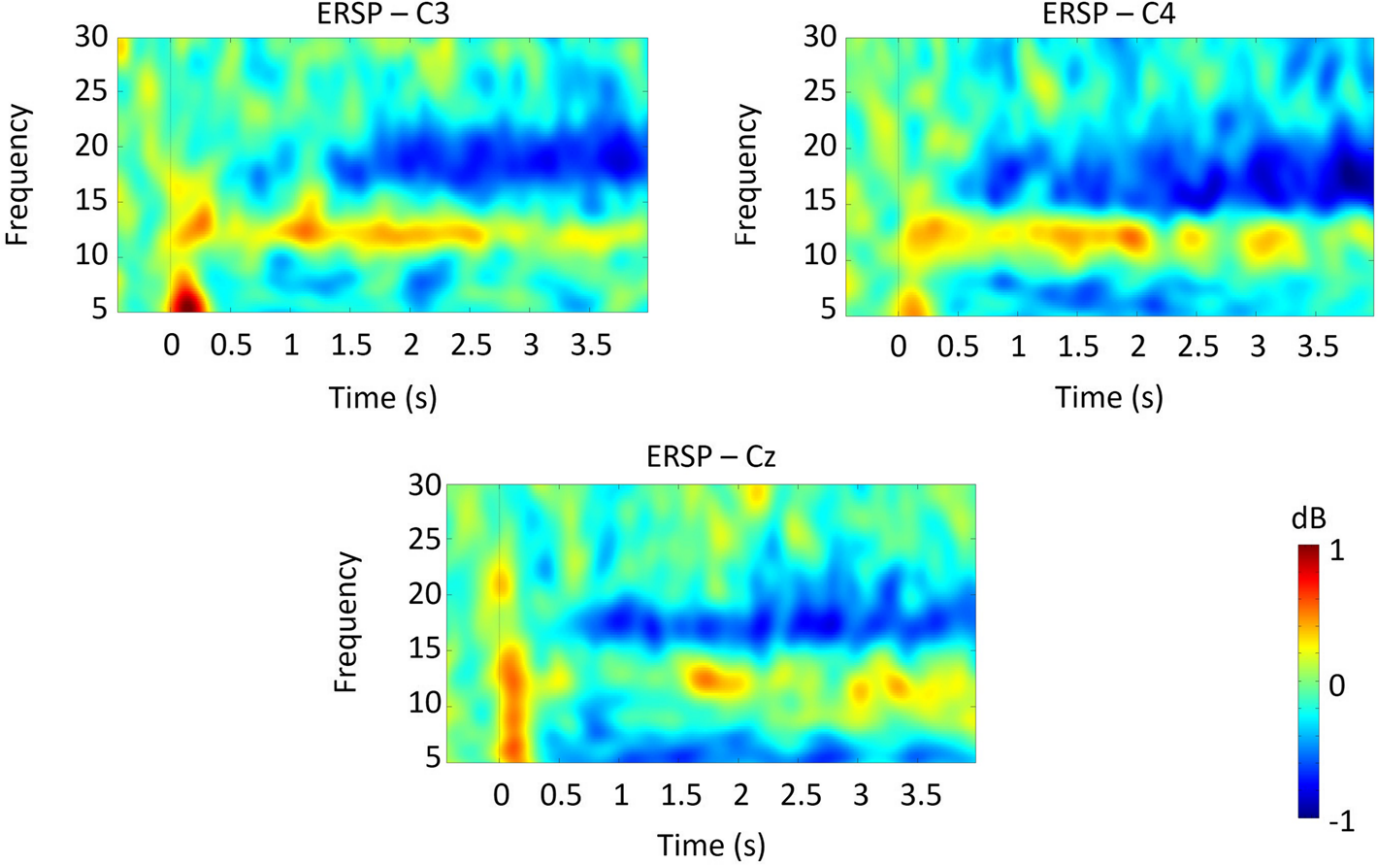
Time-frequency maps obtained for electrodes Cz, C3 and C4 for the TPMI task. Similar ERD and ERS maps were obtained for Fronto-Central and Central-Parietal electrodes

Figure 6 shows the Event Related Spectral Perturbation (ERSP) maps obtained for all subjects when the TPMI task is performed. The maps show an unexpected bimodal pattern, with a lower α synchronization pattern in addition to the high μ - low β ERD phenomenon over the imagery period.

Figure 6 depicts only the phenomenon in three selected central electrodes. However, this is also observed in Fronto-Central and Central-Parietal electrodes. The findings in the upper α and low β range may be interpreted as a result of the fact that third person imagery also involves imagery of movements, which neurons involved in the preparation of a movement may be automatically activated. Importantly, one can also observe an interesting pattern of narrow band Event Related Synchronisation (ERS) between 11 and 13 Hz.

From the analysis of these maps, it is clear that a bimodal synchronization/desynchronization pattern occurs within a narrow range frequency band. The 8–13 Hz band is most useful to identify the TPMI pattern that describes the participants’ neuronal activity.

### The neural representation of self motor imagery (participant grasping a sphere)

**Fig. 7:**
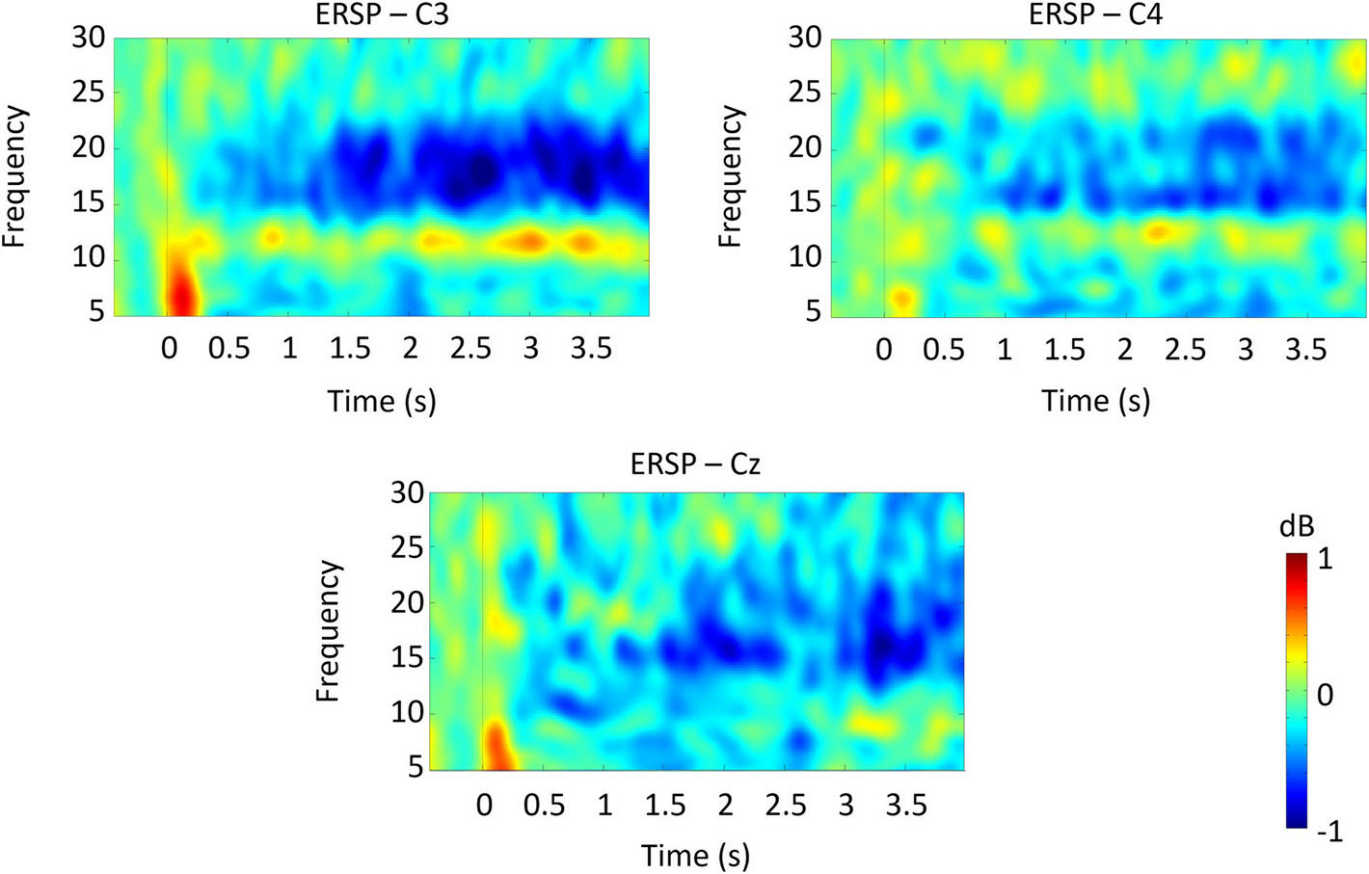
Time-frequency maps obtained using Cz, C3 and C4 electrodes for the SMI task. Similar ERD maps were obtained for Frontro-Central and Central-Parietal electrodes

Figure 7 shows the ERSP maps obtained for SMI. The maps show an ERD phenomenon similar to the TPMI task, around the low β range (which possibly occludes a similar phenomenon at the high α range). The ERS verified for SMI is still clearly observed albeit attenuated.

### Discrimination between third person and self motor imagery

**Fig. 8:**
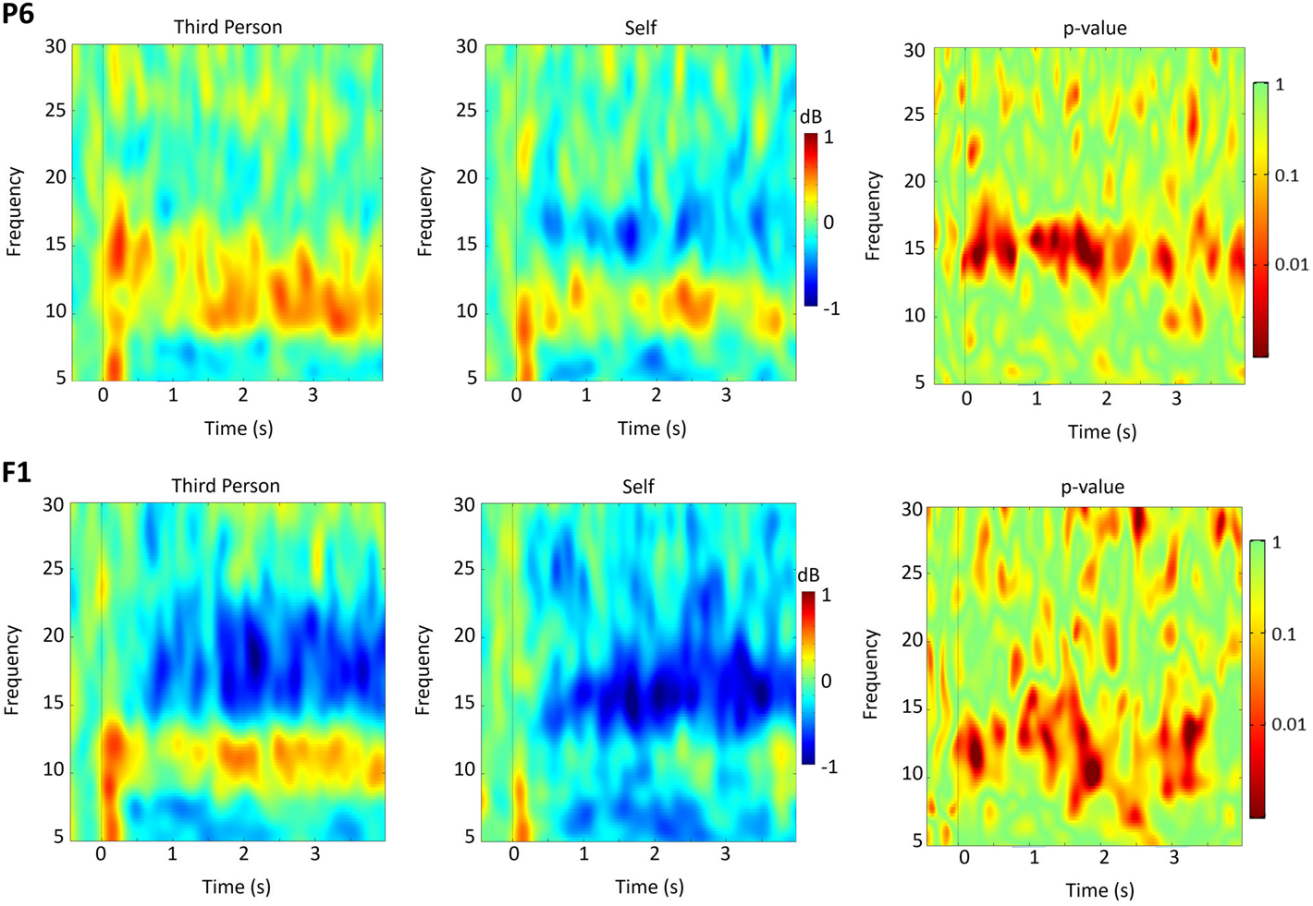
P6 (**a**) and F1 (**b**) electrodes ERSP maps obtained using a “study” on EEGLab toolbox from Matlab. The map at the left corresponds to the third person motor imagery (TPMI), while the map in the middle represents the brain response for the imagery of participant himself grasping the sphere (SMI). At the right side, it is also shown a statistical map resulting from the computation of differences between both imagery tasks, using paired t-test (note that these tests survive corrections for multiple comparisons concerning the clusters of interest)

Through a time-frequency group analysis over the EEG dataset, we found a statistically significant difference (*p* < 0.01) in the μ rhythm and low β rhythm bands. Figure 8a and b show the ERSP maps for both tasks in a frontal and a parietal electrode. We also show a map of differences between tasks graded by the significance of *p*-values, using paired t-tests. The blue band that can be seen in Fig. 6a roughly between 15 and 25 Hz represents an ERD and is more present in SMI, i.e. when the participant imagines himself grasping the sphere. On the other side, when the participant imagines the avatar grasping the sphere (TPMI), a “red” band, representing an ERS, with higher power than in SMI condition (in the case of this electrode), is evident approximately between 8 and 13 Hz. This difference is shown in the map at the right of Fig. 8a and b and it is very consistent throughout the whole imagery period.

Standard hypothesis driven statistical analysis showed no statistically significant differences in the group analysis between SMI and TPMI conditions, after Benjamini-Hochberg correction for multiple comparisons [40]. However, when these differences are analysed subject-wise, 7 of the 20 participants present statistically significant differences in different clusters. The fact that we did not find a clearly more represented cluster is evidence for the between-subject variability we see in this specific task.

Concerning the ECoG experiment, after Bonferroni correction for multiple comparisons, differences were observed between SMI-own-left and TPMI-own-left (right to the participant). The conditions SMI-own-left and TPMI-own-right (left to the participant) also showed a tendency for difference, however it does not survive for the multiple comparison correction. This difference was observed in cluster 4 (Figure 5).

### Source analysis

**Fig. 9:**
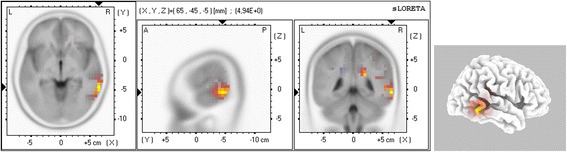
Source analysis result using sLoreta software package

The results obtained after computing the statistical test for source analysis presented in eq. 2 are shown in Fig. 9.

Source analysis showed statistically significant differences in the right hemisphere (higher activity for TPMI conditions) in Brodmann Areas (BA) 21 (maximum), 22 and 31, located in the Posterior Middle and Superior Temporal Gyrus (near the temporal parietal junction) and Cingulate Gyrus.

### Automatic discrimination between third person and self motor imagery

Importantly, we asked whether automatic statistical classification could identify which type of task (TPMI and SMI) is being performed at each instant (single trial level).

**Fig. 10:**
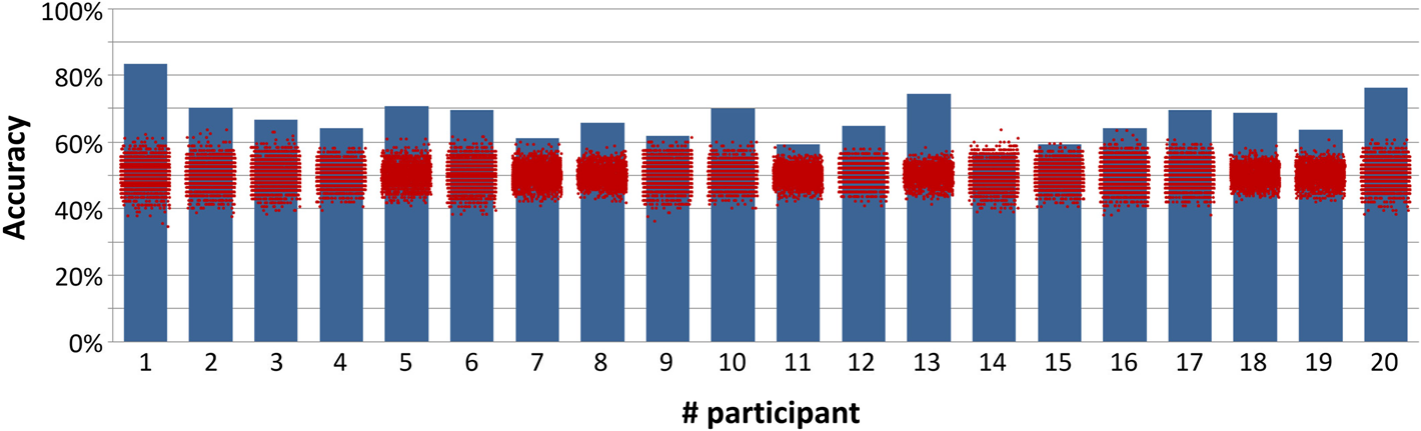
Best single-trial classification results per participant (blue bars) and permutation results for chance level comparison (red dots)

Figure 10 shows the single trial accuracy results obtained for each participant. All the participants of this study achieved between MI types classification accuracy above chance level (*p* < 0.02) according to permutation tests, which goes well beyond the previous study of Neuper et al. [24], which only discriminated against baseline. The average single trial classification accuracy for all participants was 67 ± 1% (Standard Error of the Mean – SEM – error metric for group analysis).

The classification accuracy is directly dependent of the participant performance, and it will obviously increase as the acquisition time increases (the level of experience/expertise of the participant increases).

Additionally, if instead of single trial classification, 3 trials (i.e. imagery period of 12 s instead of 4 s) are used for classification, the overall accuracy mean rises to 72 ± 2% (SEM).

**Fig. 11:**
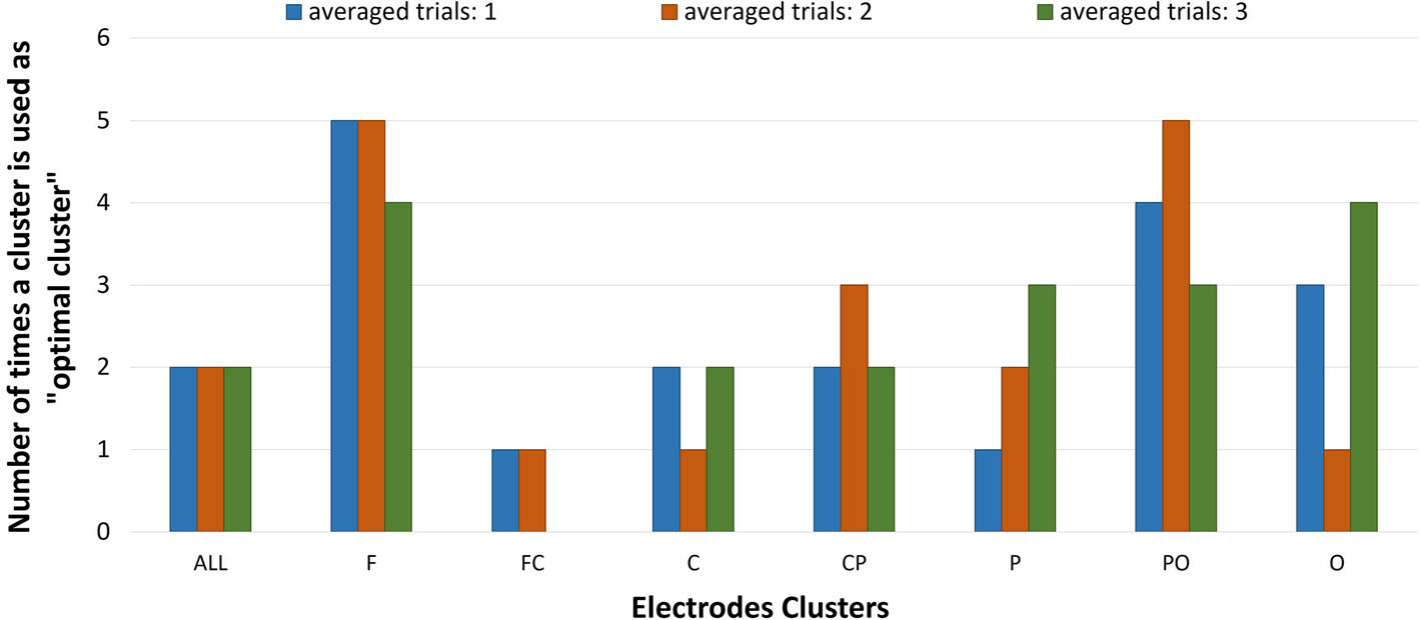
Number of times a cluster is used as “optimal cluster” for single trial and averaged (2 and 3) trials

From the clusters of electrodes used (aforementioned), the optimal cluster was defined as the cluster that gives the best accuracy for each participant. Although still with high between-subject variability, the optimal clusters confirmed to be located in the Parieto-Occipital and Frontal areas (Figure 11).

## Discussion

The goal of the present work was to study and identify the neurophysiological correlates of third person motor imagery and self motor imagery and their separability at the single trial and individual level. We showed that this was the case, which may also help pave the way for the development of a training system based on motor imagery that can help individuals to easily perceive and identify the intention of a third person. This distinction is important, because it allows to go beyond simple imitation. To test our hypotheses, we developed a motor imagery paradigm that was presented to the participants, and time-varying activation maps of their brain were analysed.

We used EEG to compare human cortical activations when a participant imagines himself or a third person grasping a sphere. The latter condition is expected to more specifically recruit the circuits that represent action understanding and intentional behaviour mirror neuron system that mainly represents non owned behaviours.

The major identified EEG patterns were related with the α rhythms (8–13 Hz, including non motor and μ related components) as well as with low β frequencies (around 17 Hz). Our observations indicate that two frequency bands of interest are present: a higher one, possibly related to motor desynchronization (low-β, 15–21 Hz) [41] and a lower one peaking around 11 Hz. It is possible that the strong low α band synchrony might be partially occluding μ related desynchronization around 13 Hz while desynchronization above that frequency was more evident. In other words, we have identified two distinguishable components in neighbouring frequencies, one with higher imagery related synchronization (possibly related to distinct frontal and posterior neural generators), and the other related to desynchronization (evoked by central motor circuits). The mechanisms underlying perception of action intention beyond simple imitation are still largely unknown, although the role of both frontal and parietal brain areas seems undisputed [42, 43] and our data suggest that they contribute to the separation of own action and third person imagery.

Accordingly, our results show that the action understanding and mirror neuron systems respond differently to the two experimental conditions, and deal with both differences and similarities underlying grasping movements of the self and others. Centro-parietal cortex was activated when both imagery tasks were performed, suggesting that both external (third person) and internal (self) motor imagery have a similar representation and activation patterns in that region (the component related to simple imitation). This region was already reported to play a key role in visuomotor control and mirror neuron system for hand actions [44–47]. Although motor activation is seen in the time-frequency maps in these central regions, that does not necessarily imply that the main differences are at these sites, in particular for the particular task distinction addressed in this study.

Key between-task differences were found in Frontal and Parieto-Occipital areas. Parieto-Occipital areas, namely high-level visual cortex (corresponding to BA 19 and 18), are related to visual memory recognition [48, 49] and visual mental imagery [50]. Frontal areas, namely supplementary motor area (SMA), anterior middle frontal gyrus and regions in the inferior and superior temporal gyrus (BA 8, 22, 46, 45, 47), are related to motor imagery [51] and learning [52]. Activation near the temporoparietal junction may reflect mirror representations for grasping and expressive movements [53–55], and attribution of intention to others [56]. Possibly, a contribution to these signals might also arise from the anterior cingulate gyrus, given the functional overlap with above mentioned regions as well as evidence to be related to self vs. others representation overlap during social perception [57, 58].

Importantly, the neurophysiological differences between third person and self imagery classified in electrodes with proximity to these regions seem to relate to different forms of α rhythms, slightly below the *μ* frequency band that is reported to be linked to motor imagery [59, 60].

Besides these general differences observed in Anterior and Posterior cortex, we observed a very defined ERD difference in the ERSP plot in P6, that might correspond to regions related to theory of mind [61]. This difference was observed slightly above 13 Hz on the low β band, possibly related to mirror-neuron type of sensorimotor processing [59, 60].

The centrally located patterns of activity we report here for imagery tasks of grasping are consistent with previous studies of imagery of reaching-to-point or reaching-to-grasp movements as well as of pointing movements [47, 62–66].

Concerning functional interpretation of our results, it is worth pointing out the conceptual framework of Bien et al. [67] who took the results of their fMRI study, in healthy subjects, as indicating that the right premotor cortex is involved in the automatic mental construction of the imitation of movements and the right middle/inferior frontal lobe in inhibition of the real execution of such imitation. So, the motor imagery is composed by those two components: imitation of movements (mental image of gesture) and the inhibition of the movement. In this line of thinking, our results are in agreement with this conceptual framework, by showing relevant patterns of brain activity which topography might arise from centro-parietal as well as middle/inferior frontal regions (which are also known to generate α activity [68–70]).

Regarding source localization of the contrast computed, maximal statistically significant differences were observed in areas related to attribution of intentions to others [56] and self/other distinction [71, 72].

Concerning the automatic discrimination between third person and self motor imagery, we consider that the resulting single trial classification accuracy was very promising. According to the obtained results, it is possible to conclude that Third Person and Self Motor Imagery use a different electrophysiological mechanism at the single individual and trial level, which allows automatic classification of the type of motor imagery. The fact that all our participants achieved statistically significant accuracy results suggests that in our case the potential curse of BCI illiteracy (inability of a subset of people to use certain BCI paradigms) is not a problem as suggested in other studies of motor imagery reporting that it could affect up to 20–30% of the participants [73]. However, the analysis is still quite participant-dependent, for instance, concerning the optimal cluster for classification. Our strategy goes beyond the previous work of Neuper et al. [24] who achieved significant classification accuracy only against baseline. Moreover, accuracies rises further if ones increase the number of trials used (from 1 to 3, above 70%) which may be a feasible strategy for the type neural state control that this type of paradigms seeks.

This was also verified when analysing the ECoG experiment with the same task. In this case, the cluster that showed statistically significant differences was Centro-Parietal, suggesting that posterior contributions may be relevant for the type of distinction investigated in this study.

In this experiment, we could also show that the differences were observed near the sensorimotor cortex when the participant was imagining her own left limb movement, which is ipsilateral to the hemisphere where the grid was placed. This suggests that the differences were more evident when this region was not active. Hence, the sensorimotor cortex is involved in both right and left TPMI.

The usage of 58 electrodes to record EEG data can be seen as a potential limitation of the application of the proposed paradigm to train the interpretation of social intentions, but this is the first step to identify the neural signals and their neural substrates, as well as to show that is possible to use these signals as markers of correct interpretation/association of intentions in social contexts. Further work should be made to design a serious game interface (as postulated by Friedrich et al., 2014 [74]) that includes the same concept used here in order to develop a potential EEG application, based on BCI, for clinical use in disorders of social cognition and action understanding such as autism. Our work seems to further favour quantitative electroencephalography approaches and their potential to identify unique electrophysiological phenotypes which can be used in neurofeedback. This is relevant because in individuals with autism, μ rhythm suppression is decreased, which is consistent with altered MNS [74]. Training children to control μ or related rhythms may lead to functional improvements and here we extend this notion to the specific notion of self vs. other movement encoding. The goal is that the feedback can be related to the specific significance of the signals being trained as also discussed by Friedrich et al., 2014 [74], and the gaming approach proposed therein. Moreover by addressing self vs. other movement, we go beyond simple imitation behavior, but in line with the rationale underlying social mirror games [74].

## Conclusion

This work assessed the neural correlates of two types of motor imagery, self motor imagery and third person motor imagery, at the single trial and individual level, with the goal to go beyond mere imitation, which is critical in autism.

Statistically significant differences were found in time-frequency, source localization and statistical classification analysis between both conditions, using electroencephalography, with a surprisingly relevant contribution of posterior and frontal sites, which are also known to generate independent α rhythms. Important differences were also seen in the temporal lobe, near the temporoparietal junction, according to the source analysis. Moreover, a considerable number of participants achieved relatively high classification and all of them achieved statistically significant classification accuracies (*p* < 0.02) in the automatic discrimination between the two types of motor imagery at the single trial level (with accuracies raising further when a few trials were added, which is relevant for state control approaches, as investigated here).

These findings, as well as the ECoG acquisition with the same task, contribute to the discussion on the distinction between different networks involved in action understanding and motor imagery beyond simple imitation and may provide new insights into the physiology of the mirror neuron system circuitry and its relation with the neural correlates that allow for decoding of the intention of others.

## Abbreviations

BA: Brodmann Area
BCI: Brain-Computer Interface
CSP: Common Spatial Pattern
EEG: Electroencephalography
ERD: Event Related Desynchronisation
ERS: Event Related Synchronisation
ERSP: Event Related Spectral Perturbation
fMRI: Functional Magnetic Resonance Imaging
MI: Motor Imagery
MNS: Mirror-Neuron System
MP: Motor Performance
PCA: Principal Component Analysis
SEM: Standard Error of the Mean
SMA: Supplementary Motor Area
SMI: Self Motor Imagery
SVM: Support Vector Machines
TPMI: Third Person Motor Imagery
